# Imprecise Medicine: BRCA2 Variants of Uncertain Significance (VUS), the Challenges and Benefits to Integrate a Functional Assay Workflow with Clinical Decision Rules

**DOI:** 10.3390/genes12050780

**Published:** 2021-05-20

**Authors:** Judit Jimenez-Sainz, Ryan B. Jensen

**Affiliations:** 1 Department of Therapeutic Radiology, Yale University School of Medicine, New Haven, CT 06520, USA; 2 Department of Pathology, Yale University School of Medicine, New Haven, CT 06520, USA

**Keywords:** BRCA2, variants of uncertain significance, DNA repair, homologous recombination, hereditary breast and ovarian cancer (HBOC), BRCAness

## Abstract

Pathological mutations in homology-directed repair (HDR) genes impact both future cancer risk and therapeutic options for patients. HDR is a high-fidelity DNA repair pathway for resolving DNA double-strand breaks throughout the genome. BRCA2 is an essential protein that mediates the loading of RAD51 onto resected DNA breaks, a key step in HDR. Germline mutations in BRCA2 are associated with an increased risk for breast, ovarian, prostate, and pancreatic cancer. Clinical findings of germline or somatic BRCA2 mutations in tumors suggest treatment with platinum agents or PARP inhibitors. However, when genetic analysis reveals a variant of uncertain significance (VUS) in the BRCA2 gene, precision medicine-based decisions become complex. VUS are genetic changes with unknown pathological impact. Current statistics indicate that between 10–20% of BRCA sequencing results are VUS, and of these, more than 50% are missense mutations. Functional assays to determine the pathological outcome of VUS are urgently needed to provide clinical guidance regarding cancer risk and treatment options. In this review, we provide a brief overview of BRCA2 functions in HDR, describe how BRCA2 VUS are currently assessed in the clinic, and how genetic and biochemical functional assays could be integrated into the clinical decision process. We suggest a multi-step workflow composed of robust and accurate functional assays to correctly evaluate the potential pathogenic or benign nature of BRCA2 VUS. Success in this precision medicine endeavor will offer actionable information to patients and their physicians.

## 1. Introduction

**Figure 1:**
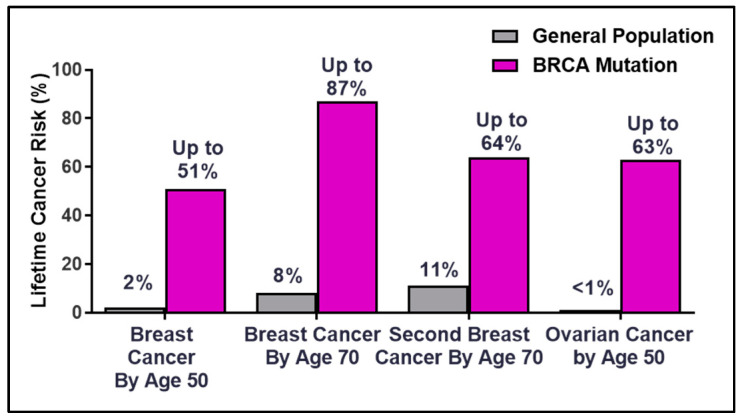
Percentages of lifetime breast and ovarian cancer risks in BRCA germline carriers.

Breast Cancer Susceptibility Gene 2 (BRCA2) was identified in 1994 at the Institute of Cancer Research by the Stratton Laboratory [1,2], only one year after the gene mapping of BRCA1 [3]. Germline mutations in the BRCA genes account for 20–60% of breast cancer cases in families with multiple cancer incidence [4]. The lifetime risk for breast and ovarian cancer rises dramatically for BRCA mutation germline carriers compared to the general population (Figure 1). The overall risk varies by family history, type and location of the mutation, age at diagnosis, parity (number of term pregnancies), environmental factors, and genetic modifiers [5,6,7,8,9,10]. The discovery of the hereditary breast and ovarian cancer (HBOC) BRCA genes in the early 1990s laid the framework for the future of precision medicine as patients became empowered with the ability to make life-changing decisions to manage and mitigate cancer risks [11,12].

Rapid advances in DNA sequencing technology coupled with public awareness and quick adoption of genetic testing for HBOC genes in genetic counseling clinics have resulted in vast database repositories of BRCA mutations and variants. While the majority of BRCA mutations are associated with breast, ovarian, pancreatic, and prostate cancers, mutations have been documented in other tumor sites such as lung and malignant melanoma.

Soon after the BRCA gene sequences were determined, Myriad Genetics initiated a vigorous intellectual property protection strategy providing them with the exclusive rights to provide all diagnostic testing. A supreme court decision in 2013 overturned Myriads’ patents and opened up the genetic testing landscape to several commercial and academic laboratories. As a result, thousands of BRCA variants have been deposited into various databases. While the majority of BRCA sequencing information has been released, Myriad still maintains a large database of BRCA variants not accessible by the public research community (https://www.wsj.com/articles/geneticists-call-on-myriad-to-share-proprietary-data-to-aid-gene-tests-11578851248, accessed on 5 May 2021).

Genetic testing of patient germline DNA and primary tumor DNA (or circulating tumor DNA (ctDNA)) has resulted in more than 30,000 genetic changes in the BRCA genes that lack functional interpretation. This knowledge gap has led to uncertainty in the ability of genetic counselors to predict cancer risk and impedes the successful clinical management of patients who may benefit from targeted therapies. International collaborations and consortiums have joined forces to design integrative approaches to combine familial and clinical data, in silico predictions, and functional analysis of BRCA2 variants of uncertain significance (VUS). However, the challenge to evaluate extremely rare VUS looms large and functional assays may be the only avenue for some patients to rely on for fundamental life-changing preventative clinical options.

In this review, we briefly cover the role of BRCA2 in HDR repair of DNA double-strand breaks (DSBs), pathogenic BRCA2 mutations and their impact on cancer risk and treatment options, database and classification methods for VUS, current clinical management strategies for BRCA2 patients, and the challenges of adopting functional analyses into clinical practice.

## 2. BRCA2 Protein, Functions, and Patient Mutations

**Figure 2:**
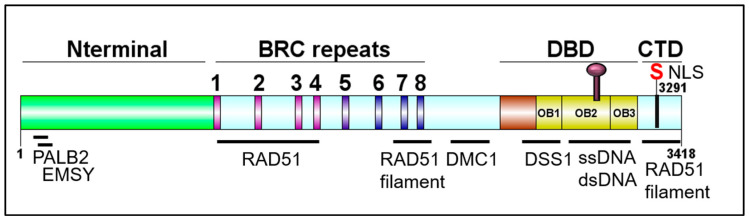
Schematic of BRCA2 domains. DBD: DNA binding domain; CTD: C-terminal domain. DOG illustrator of protein domain structure [50].

BRCA2 plays a key role in DNA damage repair via HDR, a high-fidelity repair pathway for DNA DSB repair [13,14]. In mammalian cells, DNA DSBs can be repaired by two major pathways, non-homologous end joining or HDR. HDR requires a template, usually the sister chromatid, and is thus restricted to the S or G_2_ phase of the cell cycle. If a cell encounters a DNA DSB and commits to the HDR pathway, several nucleases will resect the DNA end resulting in single-stranded DNA (ssDNA) 3’ terminated tails. The ssDNA is immediately coated with Replication Protein A (RPA) to remove the secondary structure and prevent self-annealing. BRCA2 then delivers RAD51 to the ssDNA displacing the RPA protein while simultaneously preventing RAD51 from binding to dsDNA and downregulating the ATPase activity of RAD51 [15]. These mediator activities of BRCA2 result in nucleation and stabilization of RAD51 nucleoprotein filaments on ssDNA, a necessary step preceding RAD51-dependent DNA strand invasion into a homologous donor [15,16,17,18,19,20,21]. Another interesting feature of BRCA2 is its role in replication fork protection. Utilizing the DNA fiber combing technique, several groups have found that under conditions of replicative stress, BRCA2 deficient cells undergo degradation of nascent DNA [22,23,24,25,26]. The hypothesis is that RAD51 is unable to protect the annealed nascent DNA strands at a reversed fork in the absence of BRCA2, and unchecked nucleolytic degradation by MRE11 and other resection nucleases leads to fork collapse and DNA DSBs [25]. A separation-of-function mutation located in the C-terminal domain of BRCA2 (Figure 2), S3291A, was shown to have a defect in fork protection while remaining functionally competent for HDR [25]. Described in detail elsewhere, an ever-growing list of functions has been ascribed to BRCA2, such as R loop processing, gap suppression following DNA replication, and cytokinesis [27,28,29,30,31].

The BRCA2 genomic locus is 84 kb, contains 27 exons, and is located on chromosome 13 [12]. The BRCA2 gene encodes a 3418-amino acid nuclear protein with four distinct domains: An N-terminal domain containing residues important for interactions with PALB2 and EMSY, a central BRC repeats region, a DNA binding domain (DBD) containing three tandem oligonucleotide/oligosaccharide-binding folds (OB-folds), and a C-terminal domain (CTD) (Figure 2). PALB2 (Partner and Localizer of BRCA2) mediates the interaction between BRCA1 and BRCA2, plays a role in the nuclear localization of BRCA2, aids in RAD51 loading at resected DNA breaks, and pathogenic mutations in PALB2 have been found to predispose to breast, ovarian, and prostate cancer. EMSY is a BRCA2-interacting transcriptional repressor that intriguingly is linked to sporadic breast and ovarian cancer [32,33,34,35,36]. The eight BRC repeats play critical roles in binding and regulating RAD51 loading and filament stability [37,38,39]. The CTD also functions in binding and stabilizing the RAD51 nucleoprotein filament. BRCA2 plays an important role in meiosis, and hence, binds the meiotic counterpart to RAD51, DMC1 [40]. DSS1 is a small acidic protein visible in the crystal structure of the DBD, makes several intimate contacts with BRCA2, and has been found to facilitate RPA displacement [41,42,43]. Together, all of the protein and DNA interaction domains of BRCA2 promote successful HDR and fork protection [32,35,37,38,43,44,45,46,47,48,49].

It is essential that BRCA2 localizes to the nucleus to carry out its genome integrity and tumor suppressor functions, and thus, contains several putative nuclear localization signals (NLS) [51,52,53,54]. Surprisingly, only a handful of studies have characterized both the NLS and nuclear export signals (NES) of BRCA2, and it remains unclear which specific sequences are necessary and sufficient for the proper regulation of BRCA2 nuclear/cytoplasmic trafficking [51,52,53,54,55].

In cellular models, disruption of BRCA2 leads to the accumulation of DNA DSBs, a severe decrease in HDR activity, and consequently, sensitivity to DNA damaging agents that induce replication fork stalling and/or DNA DSBs such as crosslinking agents or PARP inhibitors (PARPi) [44,56,57,58]. In addition, BRCA2 deficiency leads to micronuclei formation and centrosome amplification, ultimately contributing to genomic instability [59]. BRCA2 knockout mice are embryonic lethal, and only a handful of cell lines (VC8 (hamster); DLD1, CAPAN1, PEO1 (human)) are viable without the expression of full-length protein supporting the essential role that BRCA2 plays in early development and viability [60,61,62,63,64].

Cancer-associated mutations in BRCA2 are commonly found in the BRC repeat and OB-fold regions [9,65,66]. Population-based studies identified more than 10 BRCA2 founder mutations including: 6174delT (BRC repeat region) in Ashkenazi Jews and 7252C>T (DBD region) in the Korean and Finnish populations [67,68,69,70]. BRCA2 mutations found to increase cancer risk consist of indels, frameshifts, and nonsense mutations, ultimately leading to loss of expression or a truncated protein product. As the putative NLS signals of BRCA2 are located at the C-terminus of the protein, most truncated protein products will be mislocalized to the cytoplasm rendering the cells functionally null. Importantly, mutations that lead to loss of nuclear localization of BRCA2 are associated with HDR dysfunction and cancer predisposition [53,62].

The molecular route to tumorigenesis in BRCA2 mutant germline carriers remains an unsolved mystery. The majority of BRCA2 deficient tumors have lost the wild-type allele in a classic case of loss-of-heterozygosity (LOH), however, exceptions to the Knudson’s two-hit hypothesis rule have been described [71]. A generally accepted belief is that BRCA2 haploinsufficiency provides a chronic state of low-level genomic instability conducive to the accumulation of mutations that eventually drive the tumorigenic process. As BRCA2 functions seem necessary for cellular viability, the specific genetic background that is permissive for tumor growth is unknown. Furthermore, the genetic and pathological basis for why BRCA2 (and BRCA1) mutations lead preferentially to cancer of the breast and ovaries remains an ever-present enigma.

**Figure 3:**
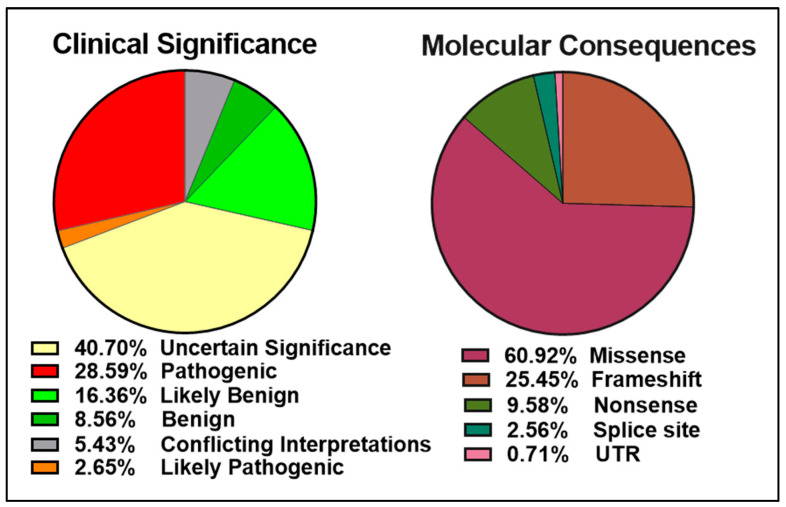
Pie charts representing the percentages of BRCA2 germline and somatic variants grouped into clinical significance (left) or molecular consequences (right) as reported in the ClinVar database (https://www.ncbi.nlm.nih.gov/clinvar/?term=BRCA2%5Bgene%5D, accessed on 10 April 2021).

The ClinVar database contains over 13,000 reports of germline and somatic BRCA2 alterations with more than 5000 VUS (Figure 3 left panel). Approximately 6000 of the 9500 BRCA2 molecular mutations reported are single amino acid changes in the 3,418-amino acid BRCA2 protein (Figure 3 right panel). Many of the mutations evaluated and reviewed as pathogenic are indeed frameshift and nonsense mutations resulting in either a truncated protein or complete lack of BRCA2 expression.

**Figure 4:**
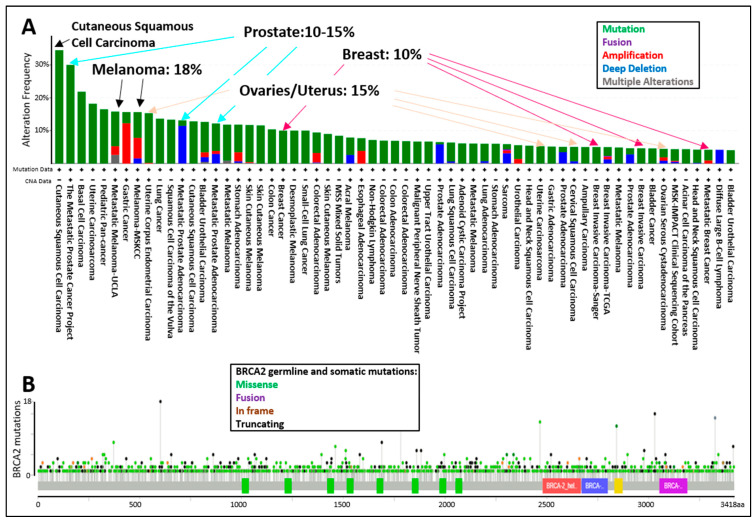
cBioPortal for Cancer Genomics display of the (**A**) percentage of BRCA2 germline and somatic alterations and (**B**) spectrum of BRCA2 germline and somatic mutations in the different protein domains. The curated set of non-redundant studies of cBioPortal for Cancer Genomics was used. Combined study with 48,081 samples and 184 studies. Graphs display BRCA2 somatic mutation frequency of 3% and BRCA2 germline mutation frequency of <0.1.

The tumor spectrum of cancers associated with pathogenic mutations in BRCA2 include: breast, ovarian, fallopian tube, melanoma, prostate, pancreatic, and lung cancers (Figure 4A). The display of all BRCA2 germline and somatic mutations described in the curated set of non-redundant studies of cBioPortal for Cancer Genomics shows that BRCA2 is altered in 10–15% of breast, ovarian, and prostate cancer cases, whereas BRCA2 is altered in 18% of melanoma cases (Figure 4A). The location of BRCA2 mutations in the dataset does not suggest specific “hot-spots” or “cold-spots” but widespread alterations across the whole gene (Figure 4B). As of 2020, a new database, HotSpotsAnnotations, applied a statistical model to detect putative hotspots on The Cancer Genome Atlas (TCGA) cancer datasets which contain 10,182 patients and 33 cancer types. In the cancer HotSpots Database, six BRCA2 mutations were identified at positions 1393, 1689, 1782, 2842, 3308, and 3342 (http://bioinformatica.mty.itesm.mx:8080/HotSpotsAnnotations/PiquinSpot.jsp?gene=BRCA2, accessed on 25 April 2021) [72]. The first three were located in the BRC domain, while the latter were mapped to the DNA binding domain. Controversy remains as some studies demonstrated no correlation, and others, a positive correlation between BRCA2 mutations in the BRC domain and ovarian cancer patient survival [73,74]. Discrepancies arise in part due to the selection of the BRCA2 mutations (missense, indels, etc.), the number of patients included, or the treatment provided. A single study in 2020 classified BRCA2 exon 10 and 11 as “cold-spots”, or more tolerant to variation [75]. However, the analysis was based on limited data, and thus overall, additional studies are needed regarding the frequency of mutation locations across the BRCA2 genomic locus.

**Figure 5:**
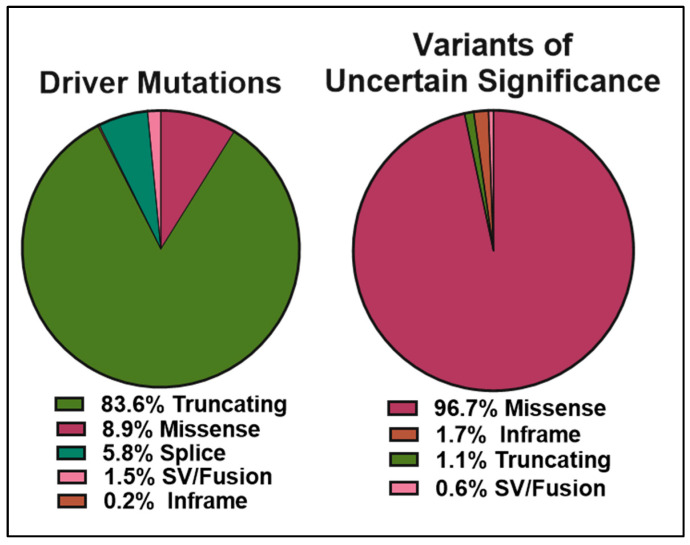
Pie charts representing the percentages of BRCA2 germline and somatic genetic variants grouped into driver mutations (left) and VUS (right) as reported in cBioPortal for Cancer Genomics database (https://www.cbioportal.org/results/cancerTypesSummary?tab_index=tab_visualize&Action=Submit&session_id=607f022de4b0242bd5d49461, accessed on 10 April 2021). The curated set of non-redundant studies of cBioPortal for Cancer Genomics was used. A total of 48,081 samples and 184 studies. The total number of driver mutations and variants of uncertain significance (1934) corresponds to 3% of the samples with BRCA2 somatic mutations and <0.1% of the samples with BRCA2 germline mutations.

The data obtained from cBioPortal indicate a total of 1934 BRCA2 germline and somatic alterations in the curated set of non-redundant studies. Of the 518 driver mutations, 83.6% are truncations (Figure 5 left panel), whereas missense mutations make up 96.7% (1369) of VUS (1416) (Figure 5 right panel). While truncating mutations in BRCA2 are clearly pathogenic in the majority of cases, the curated data highlight the need to further understand missense mutations, as they represent the most abundant VUS.

## 3. Clinical Management of Patients

**Figure 6:**
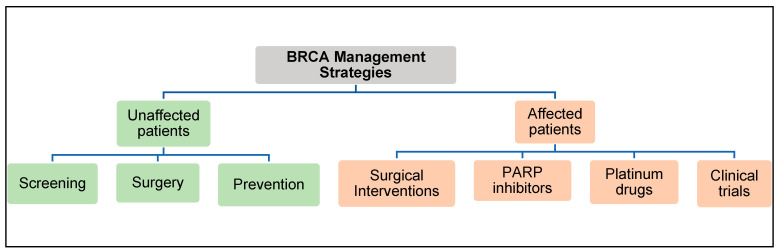
BRCA2 Management Strategies.

The American College of Obstetrician and Gynecologists (ACOG) and the American Cancer Society have created guidelines for clinicians to manage patients with BRCA2 gene mutations (Figure 6) [76,77].

Sequencing-based genetic tests to screen for BRCA mutations are offered to individuals with a family history of breast or ovarian cancer. As a risk-reduction strategy, pathogenic BRCA mutation carriers, who are cancer-free, will be informed of surgical interventions such as a double mastectomy and/or unilateral salpingo-oophorectomy. In many cases, female BRCA carriers during their early reproductive years may opt for careful surveillance in order to delay prophylactic surgeries that would prevent them from having children. In 2013, the so-called “Angelina effect” sparked an acute public awareness of genetic testing for hereditary cancer risk genes, which may have resulted in many people undergoing unnecessary testing without significant associated risk factors such as a family history of unusually high cancer incidence [78,79]. In a worst-case scenario, a recent article in the *Wall Street Journal* described a family where multiple women underwent prophylactic surgeries based on a “likely” pathogenic BRCA VUS finding that was later re-classified as a benign variant (https://www.wsj.com/articles/seven-women-in-a-family-chose-surgery-after-a-genetic-test-then-the-results-changed-11576860210, accessed on 24 April 2021). This failure of precision medicine underscores the need to cautiously interpret VUS findings and to bolster VUS evaluations with additional evidence for pathogenicity. We suggest that rigorous functional assays performed by laboratories with expertise in BRCA2 biology could provide meaningful criteria for evaluating rare VUS when familial, co-segregation, co-occurrence, and other classical genetic linkage data are lacking.

For patients who learn they are BRCA2 VUS carriers, the situation is complex and frustrating. Not only does the patient now know their BRCA2 gene is not normal (deviates from the wild-type sequence), the clinical significance of the VUS findings are rarely known, leaving the patient with unknown future cancer risk. Universally accepted standards for how to deal with BRCA VUS have yet to be established, revealing a shortcoming in our ability to make informed clinical decisions [78,79,80]. Thus, it is critical that genetic counselors have the ability to intercede and minimally inform patients of their options thus that rational choices can be made.

Patients diagnosed with cancer and for whom a germline or somatic pathogenic BRCA2 mutation is found often respond well to chemotherapy agents such as PARPi and platinum drugs. LOH of the wild-type BRCA2 allele is usually associated with a robust clinical response to these drugs but not in every case [71]. A tumor that is classified as HDR deficient is likely the best biomarker correlating with clinical response to platinum drugs and PARPi. Some patients still fail to respond to these drugs, and others may initially respond, but ultimately relapse as resistant tumor cells emerge and recolonize the tumor. Clinical trials with inhibitors such as AZD2281, 6-Mercaptopurine, and methotrexate are ongoing for BRCA2 defective ovarian tumors focused on resistance. Somatic findings of BRCA2 VUS in a tumor present a similar challenge to germline findings. In a tumor scenario, LOH of the wild-type BRCA2 allele may determine whether a patient is a good candidate for PARPi therapy. Again, if the tumor can be classified as HDR deficient, this result would provide the motivation for the use of synthetic lethal PARPi therapy. In summary, BRCA2 VUS, whether germline or somatic, presents an ongoing problem for clinical decision-making.

## 4. Curation of BRCA2 VUS

Several international groups and consortiums have joined efforts to create databases reporting BRCA2 genetic variants generated from sequencing analysis (Table 1 and Table 2). While publicly available databases are useful resources, curation of the information and classification of the variants is an ongoing challenge.

There are several commonly used online database resources that provide some interpretation of BRCA2 sequence variants (Table 2). For example, BRCA Exchange was created by the Global Alliance for Genomics and Health (GA4GH) and is the largest open-source database on BRCA variant information [83]. BRCA Exchange contains information from ClinVar, the Breast Cancer Information Core database, Leiden Open Variant Database (LOVD), several population databases, and is conveniently accessible through a smartphone app. Many of these databases, such as ClinVar and LOVD, are freely accessible and contain submissions reporting BRCA variants found in patient samples [84]. These databases display BRCA variants as expert-reviewed, interpreted, and classified by supporting evidence such as familial cases and co-segregation studies. Some of the databases display the BRCA information by molecular alteration and/or clinical significance (Table 2). The comparison of five databases with BRCA2 information shows that missense mutations are around 44–96% of the data deposited in the databases, and BRCA2 VUS make up 41–83% of ClinVar and BRCA Exchange databases. Thus, although collaborative efforts have unified the information of BRCA2 mutations, most remain unknown for cancer risk.

## 5. Methods of Classification

**Figure 7:**
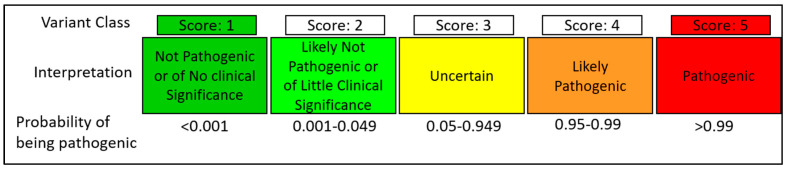
IARC proposed a classification system for BRCA2 sequence variants identified by genetic testing.

In the past decade, enormous effort has been dedicated to classifying BRCA2 VUS using a variety of clinical genetic methods and probability models. A standardized method of classifying BRCA2 variants was created by the International Agency for Research on Cancer (IARC) and the American College of Medical Genetics and Genomic (ACMG) based on a 5-tier system with a multifactorial likelihood model [85,86,87] (Figure 7). Multifactorial likelihood models describe the odds in favor of causality or neutrality based on analysis of personal and family history of cancer and co-segregation of the variant with disease in pedigrees [88]. Several genetic risk prediction models have been created based on familial and personal history. These genetic risk models include: BRCAPRO [89], IBIS [90], and BOADICEA [91]. Several multifactorial models include pathology or immunohistochemistry analysis of the tumors together with the cancer family history [92,93,94], and other models with co-segregation studies consider gender, genotype, and onset age of cancer [95].

Computational methods have been developed to help predict amino acid changes in protein function [96,97,98]. Polyphen2, SIFT, Align-GVGD, HCI database, GeneSplicer, and Human Splicing Finder are in silico online tools that can be utilized to evaluate sequence conservation and biochemical properties of changed amino acid residues in a clinical variant (see [99]) [100]. The posterior probability model combines sequence conservation and biochemical properties of the amino acid residue with the likelihood ratios [101].

Models hold the most utility in BRCA2 variants where the frequency and/or linkage analysis of the variant is high. However, rare variants (allele frequency < 0.01) are impossible to classify due to a lack of statistical power. Another layer of complexity is that the guidelines governing BRCA2 variant classification change very rapidly necessitating reinterpretation and leading to debate. Finally, if the clinical, genetic, and in silico data for BRCA2 VUS are not deposited in a freely accessible database, it becomes an almost impossible barrier for researchers and clinicians to overcome. In summary, functional studies accompanied by clinical annotation, and the aforementioned tools, may improve classification of BRCA2 variants.

## 6. Functional Studies

BRCA2 is a large and complex protein, and the literature to date has ascribed multiple functions to the protein. Loss of one particular activity does not necessarily directly link to cancer risk, and thus, evaluation of germline variants by a single assay should be interpreted carefully and in combination with clinical and familial data. In contrast, somatically mutated BRCA2 variants identified by sequencing of tumor DNA may correlate well with functional assays designed to measure chemotherapeutic response. For example, confirmation of tumor HDR deficiency is clinically actionable by stratifying patients for treatment with PARPi or platinum agents. Multiple studies have validated that in vitro cell culture models of BRCA2 deficiency respond in a similar manner to patients who receive PARPi therapy [102,103,104]. Of course, the genetic background of the patient, immune system makeup, prior treatments, tumor site, and other factors will impact the ultimate therapeutic benefit to patients.

The low frequency of rare VUS coupled with limited or no family history of cancer can make the correct evaluation of BRCA2 pathogenic classification very challenging. We and others have developed numerous biological and biochemical assays to assess the consequences of BRCA2 variants on protein function. These assays require high specificity and sensitivity to complement genetic linkage and epidemiological classification methods mentioned above successfully.

Functional complementation assays in BRCA2 deficient hamster, mouse, and human cells have been used extensively by several groups [65,67,105,106,107,108,109,110,111,112,113,114,115,116,117]. These assays have been documented in-depth in a previous review [65] but include the following: The DR-GFP reporter assay designed to measure gene conversion, yeast recombination assays, centrosome amplification, drug sensitivity assays, mouse ES cell complementation, the SyVal human DLD1 cell model (introduction of variants by gene targeting of BRCA2), nuclear localization, protein-protein, and protein-DNA interactions, and RNA splicing and mRNA maturation assays utilizing transcript analysis and minigenes [30,32,53,54,57,58,61,105,106,107,110,111,112,113,115,116,117,118,119,120,121,122,123,124,125,126]. Recently, a high-throughput functional evaluation termed the MANO-B method was used to measure the sensitivity of BRCA2 variants to PARPi in a mixed population of cells containing several BRCA2 variants. This large-scale method demonstrated a high correlation with IARC, ClinVar, and Align-GVGD classifications of BRCA2 pathogenic variants [127]. A highly useful variant analysis system was developed by the Sharan group in 2003 using a mouse embryonic stem (ES) cell-based complementation assay (functional BRCA2 VUS will rescue the lethality of a conditional BRCA2 allele). In addition to utilizing the endogenous BRCA2 promoter, this system has the advantage of maintaining the exon/intron structure of BRCA2 (the entire genomic region on a bacterial artificial chromosome (BAC) is utilized for complementation), and thus, has the ability to analyze splice variants in addition to variants within the open reading frame. BRCA2 VUS that provide viable complementation can be further tested for DNA damage sensitivity, HDR by the DR-GFP assay, or subtle changes in genomic integrity to identify hypomorphic alleles [47,105,106,107,111]. Finally, in 2018, the Shendure lab published a groundbreaking approach to analyze VUS by mutating each individual base in the RING and BRCT regions of BRCA1 utilizing a CRISPR-based technique termed saturation genome editing [128]. The approach relies on the lethality of human HAP1 cells (a unique haploid human cell line) upon the expression of a non-functional BRCA1 allele. Saturation genome editing analysis of BRCA1 variants appears to correlate well with clinically annotated variants, and this scalable approach should be applicable to other HDR genes critical for cell viability such as BRCA2, PALB2, and the RAD51 paralogs.

In total, while functional assays have been instrumental in the classification of BRCA2 VUS where familial and genetic information is scarce, clinical geneticists have been slow to incorporate lab-based results into counseling decisions for patients. Lack of standardization and uniformity, assays that can only measure specific regions of the protein, low throughput, uncertainty as to which functions of BRCA2 are necessary and sufficient for tumor suppression, and discordance between functional assay results with known variants of clinical significance have all likely contributed to the slow adoption. Furthermore, as with any clinical diagnostic assay, the risk of false negatives or false positives must be carefully addressed. However, in situations where rare VUS lack any clear genetic linkage data, we suggest that functional assays should be considered if used judiciously and with full disclosure.

**Figure 8:**
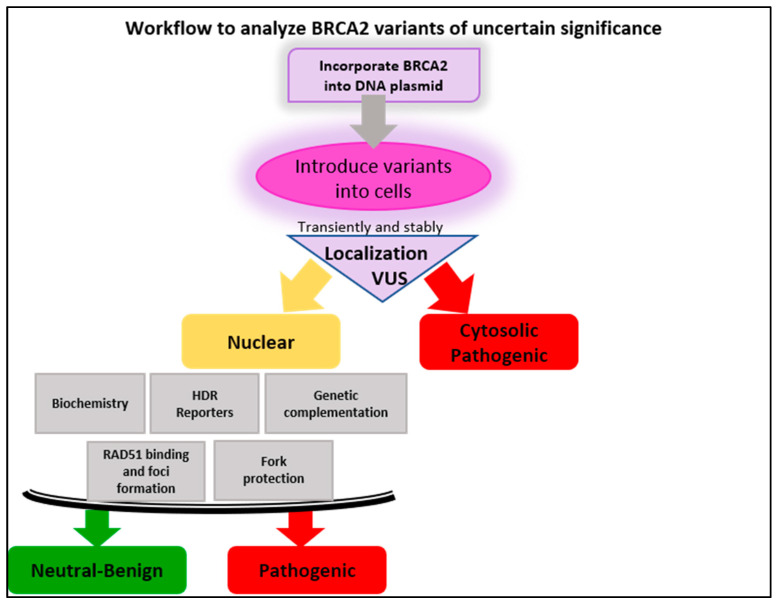
Workflow to analyze BRCA2 variants of uncertain significance.

In our laboratory, we have designed a simple multi-step workflow to cover many BRCA2 functions described to date. We have taken advantage of biochemical, genetic, cellular, and molecular complementation analysis to investigate the functionality and cellular localization of the BRCA2 protein (Figure 8). To do so, we incorporate each missense VUS into a mammalian expression vector placing the BRCA2 cDNA in frame with an N-terminal tandem maltose-binding protein (2XMBP) tag, which provides robust expression and stability. We have further demonstrated that the 2XMBP tag does not interfere with any of the cellular functions of BRCA2 [15]. The constructs are stably integrated into the human DLD1 BRCA2^−/−^ cell line for cell-based and genetic evaluation [61]. Constructs containing BRCA2 variants are also introduced into facile human cell models such as 293T cells to assess protein-protein interactions and cellular localization. Finally, we perform complementation studies in immortalized human MCF10A breast epithelial and FTSEC (fallopian tube secretory epithelial) cells, relevant to breast and ovarian cancer, respectively, in which we have introduced an inducible shRNA against BRCA2. In contrast to CRISPR, or gene-targeted knockouts of BRCA2, besides being lethal in most cells, a conditional shRNA system for BRCA2 has the advantage of acute depletion in an otherwise genetically stable background. In the near future, we plan to take advantage of CRISPR knock-in technology to create conditional BRCA2 alleles at the endogenous loci.

The advantage of using a human cell model (e.g., DLD1 BRCA2^−/−^) that tolerates a genetically BRCA2 null background is that we can evaluate pathogenic BRCA2 variants directly. Cellular complementation systems that rely on viability are limited to benign, or in some cases, hypomorphic BRCA2 alleles for further analysis as pathogenic variants do not give rise to clones. While we acknowledge that our CMV-driven cDNA complementation system forgoes expression by the endogenous promoter, evaluation of several hundred stable cell clones has revealed little difference in complementation ability due to variability in expression levels. Using our approach, we have identified multiple missense mutations predicted to be pathogenic that are mislocalized to the cytoplasm (manuscript in preparation). This unexpected observation was only possible through the expression of full-length BRCA2 proteins in the DLD1 BRCA2^−/−^ cell line. The phenotypic consequences of pathogenic BRCA2 allele cellular localization significantly impact our workflow decision process of whether further biochemical workup is warranted. A further advantage of our ability to interrogate deleterious BRCA2 alleles is that considerable uncertainty still exists as to which functions of BRCA2, HDR versus fork protection, are critical for chemotherapeutic response in patients. In summary, the potential for biological insight derived from analyzing separation-of-function alleles, and the capability to directly measure drug sensitivity for somatically mutated BRCA2 alleles in patient tumors, highlight the importance of our approach.

Our workflow for BRCA2 VUS evaluation (Figure 8) begins at BRCA2 localization by immunofluorescence. At this decision point, if BRCA2 is cytosolic, we conclude pathogenicity as cytoplasmic localization precludes any ability of BRCA2 to carry out its necessary genomic integrity functions in the nucleus. It remains unclear how and why certain BRCA2 missense VUS are retained in the cytosol, and this is an active research area in our laboratory. If BRCA2 is nuclear, we analyze biochemically and genetically the functionality of BRCA2 VUS. To do so, we leverage our ability to purify the full-length human BRCA2 protein [15,38] and interrogate altered biochemical functions such as DNA substrate and RAD51 binding, RAD51 filament stabilization, and stimulation of RAD51-dependent DNA strand exchange activity. In parallel, our cell-based models allow us to evaluate drug sensitivity (e.g., PARPi and crosslinking agents), RAD51 foci, fork protection, HDR functionality by DR-GFP assay, and gene targeting ability (Cas9/mClover LMNA assay).

Our approach complements and extends prior functional characterization of BRCA2 VUS by providing an exhaustive genetic and biochemical workup of pathogenic alleles. Pathogenic BRCA2 missense variants, in particular, present a rich source of information into the underlying biological functions of BRCA2 and can be leveraged in a “reverse translational” approach to decipher which specific attributes of BRCA2 are necessary for HDR, chemotherapeutic response, and tumor suppressor functions.

While many functional assays exist to examine HDR or fork protection activities, no specific test for the actual tumor-suppressive functions of BRCA2 has been developed. The lack of a definitive in vitro or in vivo model for the cancer-promoting activity of BRCA2 poses an issue when one considers the evaluation of germline VUS to predict future cancer risk. How does one decide which functional assay is critical for the long-term effects of BRCA2 haploinsufficiency leading to the eventual outgrowth of a clinically diagnosable cancer? The oncogene field has developed multiple approaches to interrogate the tumorigenic potential of specific gain-of-function mutations in genes such as kinases (e.g., SRC) or GTPases (e.g., RAS) including: foci assays, anchorage-independent growth in soft agar, genetically engineered mouse models (GEMM), or tumor xenograft studies. To date, no equivalent experimental approach exists for either BRCA gene. Hopefully, in the future, we will be able to parse out the exact functions of BRCA2 that are required to spark tumorigenesis, and this knowledge can be adapted into an assay that will accurately quantify the tumorigenic capacity of BRCA2 variants.

## 7. Conclusions and Future Directions

In the future, the volume of BRCA2 VUS findings will undoubtedly increase as sequencing technology becomes more economical, higher throughput, and adopted into clinical practice as a routine diagnostic. Patients, clinicians, and researchers will hopefully demand more open access to this information, with appropriate privacy controls in place, thus that VUS have a better chance of being correctly classified. The end goal of functional analysis is to derive a highly sensitive, specific, and predictive score that can be integrated with prior methods to correctly classify the large number of BRCA2 VUS and unmask both cancer risk and response to chemotherapeutics.

The current diversity and non-standard resources and databases for deciphering BRCA2 VUS information is a constant challenge for physicians and genetic counselors. Without proper curation and standardized guidelines, clinical decisions surrounding VUS are complex at best and irresolvable at worst. To improve classification efforts, a universal workflow could be adopted with agreed-upon expert guidelines to evaluate BRCA2 variants in a systematic fashion. These universal guidelines will need to escalate quickly as VUS are rapidly clogging the variant pipeline in step with clinical genetic testing panels and sequencing technology. Commercial companies such as Myriad, Ambry, deCODE, and Labcorp are adding to this VUS pipeline, and public awareness has further increased findings of VUS as more and more people seek out their own genetic information.

The collective sequences of BRCA variants are being centralized in databases such as BRCA Exchange and are growing exponentially. Just a few days before the submission of this review, BRCA Exchange announced on social media that the number of BRCA variants in their database would increase from 40,000 to over 60,000. The pace of VUS findings concurrent with inconclusive assessments of cancer risk or potential response to targeted therapy will undoubtedly accelerate in the future. Hence, the need for functional clarification intensifies.

We conclude that rationale, rigorous, and statistically sound functional analyses of BRCA2 VUS will unleash the current lack of clinical significance for thousands of rare VUS. Although laborious, biochemical and cellular functional assays have the potential to correctly assign a pathogenic or benign classification to a BRCA2 VUS. Functional information combined with familial, co-segregation, co-occurrence, and other classical genetic epidemiological data will reveal the full catalog of BRCA2 pathogenic mutations. Additionally, we will continue to leverage patient-derived BRCA2 pathogenic variants to enhance our understanding of the molecular events that lead to tumorigenesis. Our long-term goal is to remove the frustration associated with clinical BRCA2 VUS findings such that patients feel empowered with clinically actionable information fulfilling the modern promise of delivering precision medicine.

## Figures and Tables

**Table 1:** International groups investigating BRCA mutations in various populations.

Study/Consortium	Description
CIMBA	Consortium of investigators of modifiers of BRCA1/2 was established in 2005
PROSE	Consortium in the prevention and observation of surgical endpoints [81]
ENIGMA	International consortium of investigators to determine the clinical significance of sequence variants in BRCA1, BRCA2 and other known or suspected breast cancer genes (www.enigmaconsortium.org, accessed on 15 April 2021) [82]
EMBRACE	Study aims to create a register of BRCA1 and BRCA2 families with defects in other genes to find out the cancer risk in these associations [10]
IBCCS	International BRCA1/2 carrier cohort study: purpose, rationale, and study design (https://breast-cancer-research.biomedcentral.com/articles/10.1186/bcr93, accessed on 15 April 2021)
Breast Cancer Linkage Consortium	International data sharing platform established in 1989 to collect families with breast cancer by linkage analysis
PROMPT	Prospective Registry of Multiplex Testing for patients and their families (www.promptstudy.org, accessed on 15 April 2021)

**Table 2:** Clinical Significance Databases with BRCA2 mutations (Updated April 2021).

BRCA2 Databases	Total Variants	Variants of Uncertain Significance	Missense Variants	Pathogenic Variants
Breast Cancer Information Core (BIC) (https://research.nhgri.nih.gov/bic/, accessed on 15 April 2021)	14,914	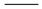	7156 (48%)	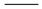
Leiden Open Variant Database (LOVD) (https://www.lovd.nl, accessed on 15 April 2021)	920	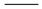	871 (94.6%)	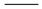
Catalogue of Somatic Mutation in Cancer (COSMIC) (https://cancer.sanger.ac.uk/cosmic, accessed on 15 April 2021)	2729	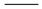	1548 (56.72%)	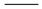
BRCA Exchange (https://brcaexchange.org, accessed on 15 April 2021)	20,751	16,649 (83%)	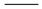	2672 (13%)
ClinVar (https://www.ncbi.nlm.nih.gov/clinvar/, accessed on 15 April 2021)	13,109	5381 (41%)	5756 (44%)	3780 (29%)

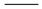
 Data unavailable.
